# Prognostic Nomogram for patients undergoing radical Pancreaticoduodenectomy for adenocarcinoma of the pancreatic head

**DOI:** 10.1186/s12885-021-08295-5

**Published:** 2021-05-27

**Authors:** Chao Wu, Sheng Zhong Hou, Zuowei Wu, Xing Huang, Zihe Wang, Bole Tian

**Affiliations:** Department of Pancreatic Surgery, West China Hospital, Sichuan University, No. 37 Guoxue Alley, Chengdu, Sichuan Province China

**Keywords:** Adenocarcinoma of the pancreatic head, Pancreaticoduodenectomy, Prognostic, Nomogram

## Abstract

**Background:**

Radical pancreaticoduodenectomy is the most common treatment strategy for patients diagnosed with adenocarcinoma of the pancreatic head. Few studies have reported the clinical characteristics and treatment efficacies of patients undergoing radical pancreaticoduodenectomy for adenocarcinoma of the pancreatic head.

**Methods:**

A total of 177 pancreatic head cancer patients who underwent radical pancreaticoduodenectomy and were pathologically confirmed as having pancreatic ductal adenocarcinoma were screened in the West China Hospital of Sichuan University. The multivariate analysis results were implemented to construct a nomogram. The concordance index (c-index), the area under the curve (AUC) and calibration were utilized to evaluate the predictive performance of the nomogram.

**Results:**

The prognostic nutritional index (PNI), the lymph node ratio (LNR) and the American Joint Committee on Cancer (AJCC) staging served as independent prognostic factors and were used to construct the nomogram. The c-indexes of the nomogram were 0.799 (confidence interval (CI), 0.741–0.858) and 0.732 (0.657–0.807) in the primary set and validation set, respectively. The AUCs of the nomogram at 1 and 3 years were 0.832 and 0.783, which were superior to the AJCC staging values of 0.759 and 0.705, respectively.

**Conclusions:**

The nomogram may be used to predict the prognosis of radical resection for adenocarcinoma of the pancreatic head. These findings may represent an effective model for the developing an optimal therapeutic schedule for malnourished patients who need early effective nutritional intervention and may promote the treatment efficacy of resectable adenocarcinoma of the pancreatic head.

**Supplementary Information:**

The online version contains supplementary material available at 10.1186/s12885-021-08295-5.

## Background

Pancreatic cancer is an extremely aggressive malignancy and has a poor prognosis worldwide [1]. Although surgical resection is a therapy implemented to treat pancreatic cancer, the rates of mortality remain high, and the 5-year survival rate is only 10–20% [2, 3]. Consequently, it is vital to discern a postoperative prognostic biomarker that could assess the risk stratification of patients and help develop an optimal therapeutic schedule. Some studies have disclosed clinical characteristics, such as resection margins, the PNI, the LNR, portal vein invasion and tumor differentiation, utilized to discriminate treatment outcomes in patients with pancreatic cancer [4–8]. Additionally, only a single indicator was used to assess postoperative survival for pancreatic cancer in those studies. For this reason, we discern some indicators that serve as prognostic markers that influence the postoperative outcome of pancreatic cancer.

The prognostic nutritional index (PNI) was first identified as a prognostic marker for patients with gastrointestinal cancer and calculated from the serum albumin level and total lymphocyte count [9]. Kanda and colleagues disclosed that the PNI was associated with overall survival (OS) and that it may be a predictor with moderate accuracy in resectable pancreatic cancer. Additionally, Lee and colleagues revealed that the PNI may be a prognostic marker for all stages of pancreatic cancer [10]. These studies found that the PNI is expected to act as a surrogate marker for preoperative assessments of the nutritional and immunological status.

The lymph node ratio (LNR) is the ratio of the number of positive lymph nodes to the total number of lymph nodes dissected during surgery [11, 12]. Previous studies have unveiled that the LNR may be a sensitive indicator of OS in patients with pancreatic cancer [13–16]. The aim of this work is to evaluate the prognostic influence of the LNR and PNI on survival in patients with adenocarcinoma of the pancreatic head undergoing radical pancreaticoduodenectomy.

## Methods

### Patients

This study was approved by the Ethical Review Committees of Sichuan University and was performed in accordance with the ethical standards and according to the Declaration of Helsinki. We retrospectively collected data from 316 patients with supposed pancreatic head cancer who were admitted to the West China Hospital of Sichuan from July 2014 to June 2017. Patients with any of the following characteristics were excluded from this study: pathologically confirmed not to have adenocarcinoma (*n* = 79), adenocarcinoma of the pancreatic head with distant metastasis (*n* = 23) or lost to follow-up after not more than 1 month (*n* = 37). Adenocarcinoma of the pancreatic head patients underwent pancreaticoduodenectomy and systematic lymphadenectomy without peritoneal dissemination or distant metastases. Finally, 177 patients with adenocarcinoma of the pancreatic head were incorporated in the study. For further analysis, the discerned patients were randomly divided into a primary set (*n* = 89) and a validation set (*n* = 88). The follow-up time was more than 3 years.

### Data collection

In resectable adenocarcinoma of the pancreatic head patients, related characteristics, such as age, sex, serum albumin, total lymphocyte count, initial serum level of carcinoembryonic antigen (CEA), preoperative carbohydrate antigen 19–9 (CA19–9), postoperative adjuvant systemic chemotherapy, and TNM stage, were screened through the electronic medical records. The PNI value was calculated as 10 × serum albumin (g/dL) + 0.005 × total lymphocyte count (/mm3) in peripheral blood [17]. In accordance with the American Joint Committee on Cancer (AJCC) 8th edition guidelines, resectable pancreatic head cancer patients were sorted into diverse stages. OS was delimited as the phase from the time of diagnosis until death.

### Statistical analysis

Survival distributions were estimated by the Kaplan-Meier method and log-rank test to compare the categorical variables of the primary set and validation set. Univariate and multivariate analyses were performed by using the Cox proportional hazards regression model. The multivariate analysis results were implemented to construct a nomogram. The concordance index (c-index), the area under the curve (AUC) and calibration were utilized to evaluate the predictive performance of the nomogram. Decision curve analysis (DCA) was implemented to evaluate the predictive power of the nomogram. A value of *P* < 0.05 was considered statistically significant. Statistical analyses were performed using SPSS V26.0 (SPSS Inc.) and R software v4.0.2 (R Foundation for Statistical Computing, Vienna, Austria).

## Results

### Patient characteristics

The clinical characteristics of adenocarcinoma of the pancreatic head patients who underwent radical pancreaticoduodenectomy are shown in supplement 1. A total of 61.5% of the patients were male. The CA19–9 level was elevated in 82.5% of pancreatic head cancer patients, and 65.5% of pancreatic head cancer patients who underwent radical pancreaticoduodenectomy had grade I/II disease according to the AJCC 8th edition guidelines. Approximately half of the pancreatic head cancer patients had elevated CEA levels at diagnosis.

### Analysis of risk factors for pancreatic head cancer

The univariate analysis and the multivariate analysis showed that the PNI, LNR, and TNM 8th edition guidelines were associated with OS in the primary set (Table 1) and in the validation set (Table 2). Namely, the multivariate analysis revealed that the PNI (HR 0.51; 95% CI, 0.273–0.952, *P* = 0.034), LNR (HR 2.543; 95% CI, 1.052–6.148, *P* = 0.038), and TNM 8th edition guidelines (HR 1.948; 95% CI, 1.351–2.810, *P* < 0.001) were independent factors related to OS in the primary set. Similarly, the PNI (HR 0.398; 95% CI, 0.217–0.729, *P* = 0.003), LNR (HR 4.087; 95% CI, 2.065–8.090, *P* < 0.001), and TNM 8th edition guidelines (HR 2.786; 95% CI, 1.939–4.003, *P* < 0.001) performed by the multivariate analysis also remained independent variables related to OS in the validation set.

**Table 1:**
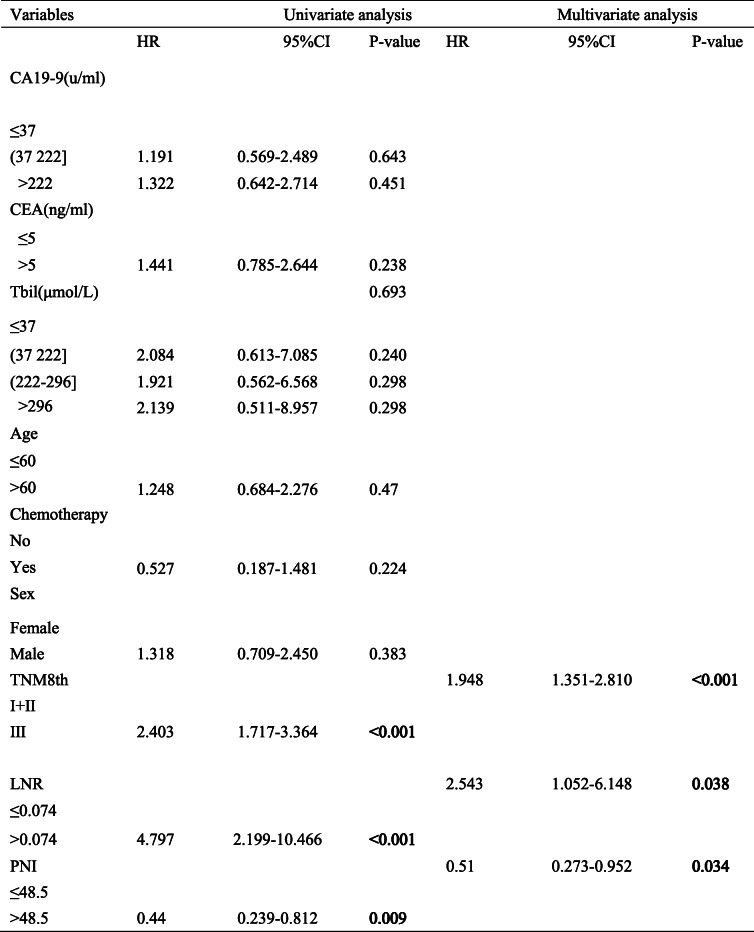
Univariate and multivariate analysis of overall survival in Primary set.

**Table 2:**
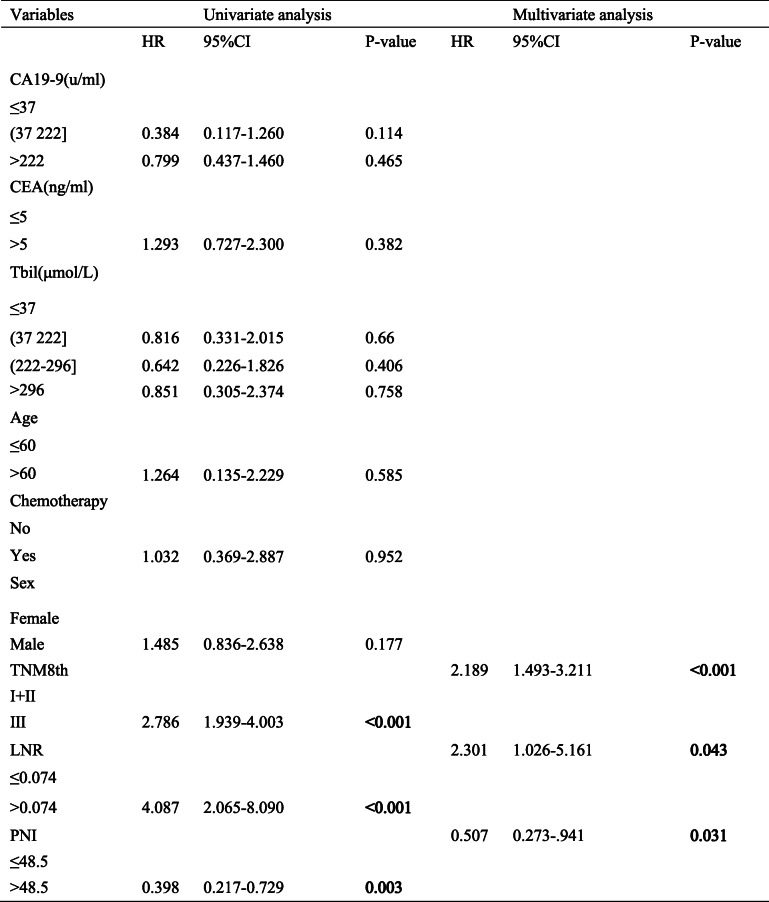
Univariate and multivariate analysis of overall survival in Validation set.

### Construction of the nomogram

**Fig. 1:**
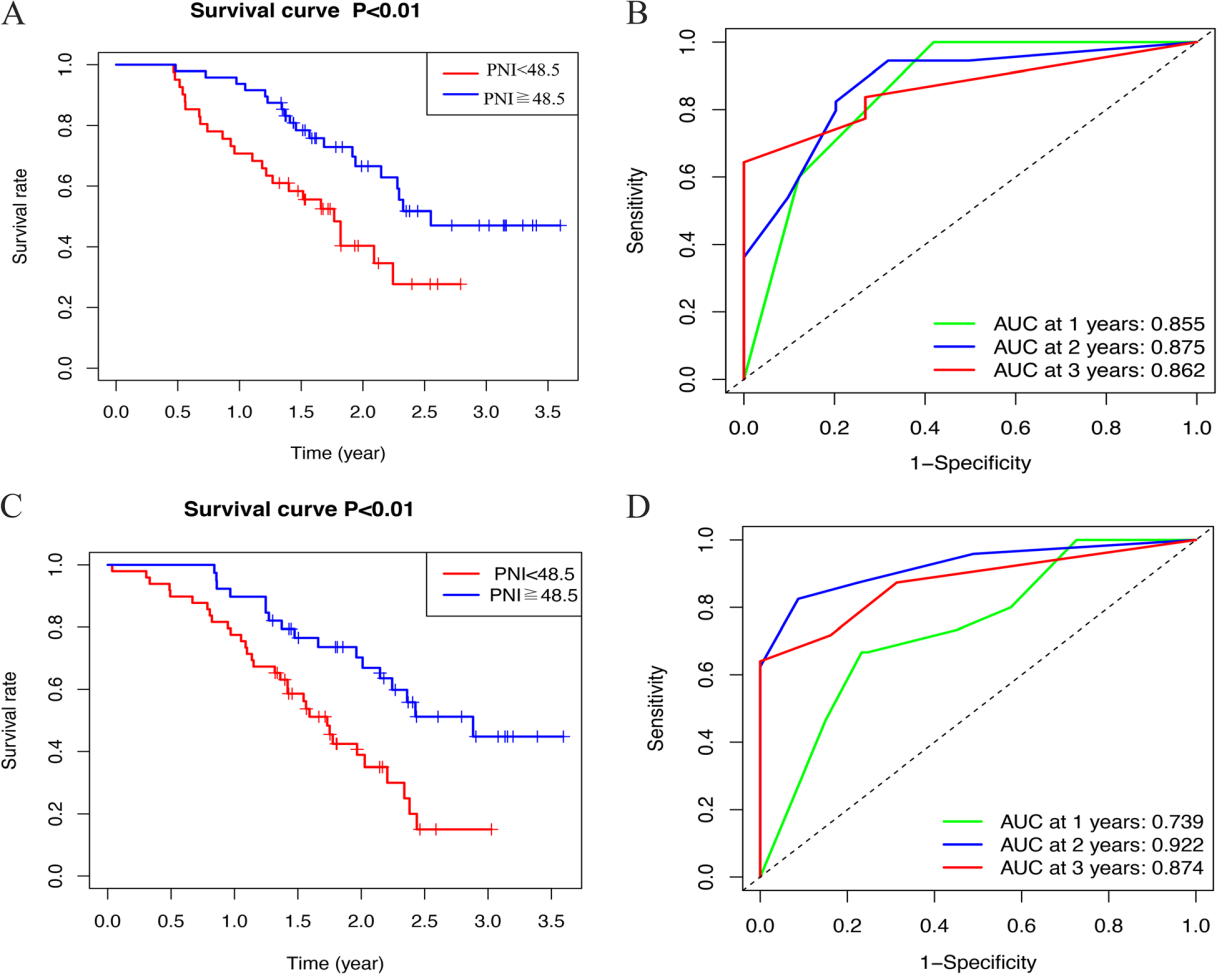
The Kaplan–Meier analysis and performance of PNI in predicting 1-and 3-year prognosis respectively in primary set (**ab**) and validation set (**cd**)

**Fig. 2:**
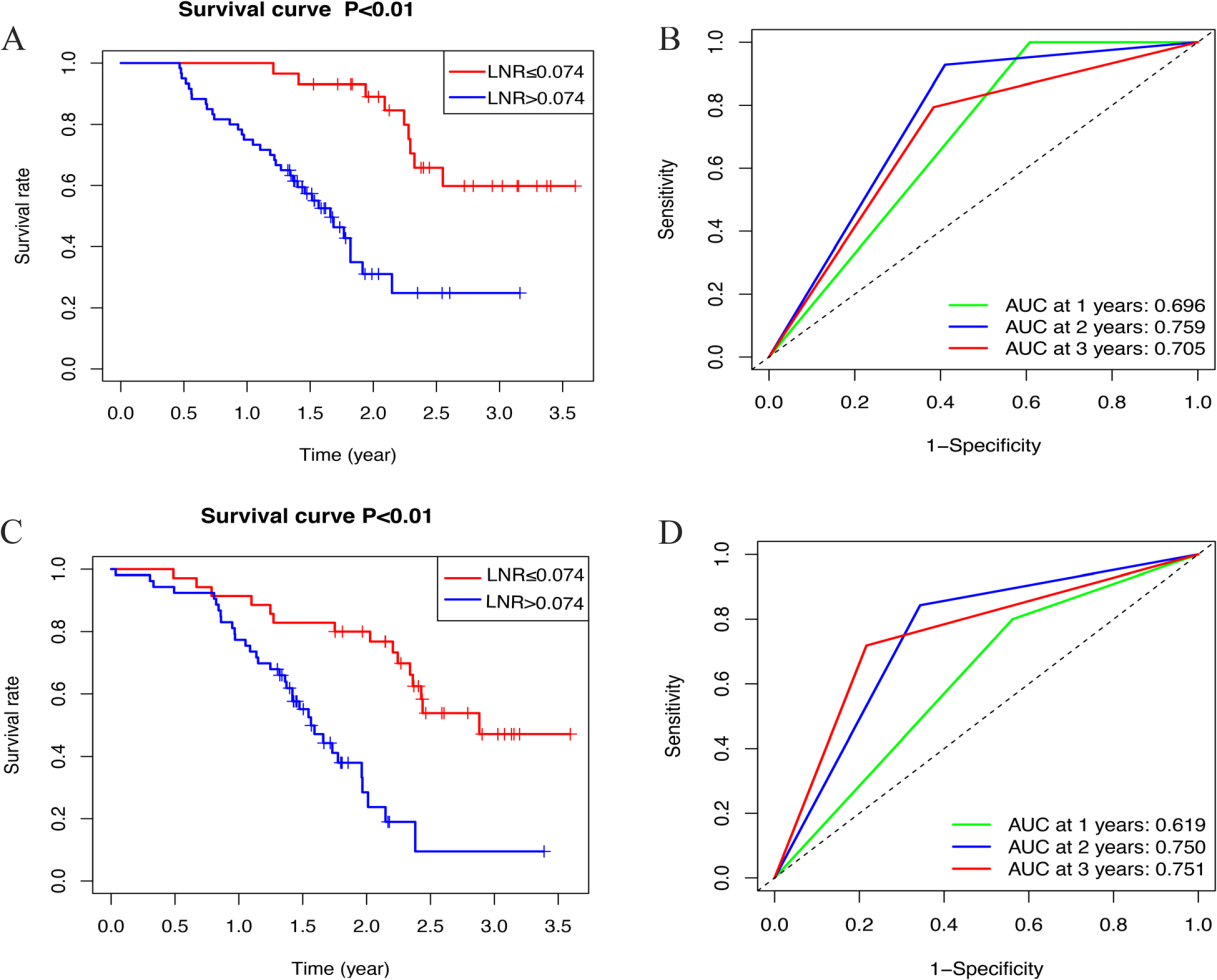
The Kaplan–Meier analysis and the predictive power of LNR in predicting 1-and 3-year prognosis respectively in primary set (**ab**) and validation set (**cd**)

**Fig. 3:**
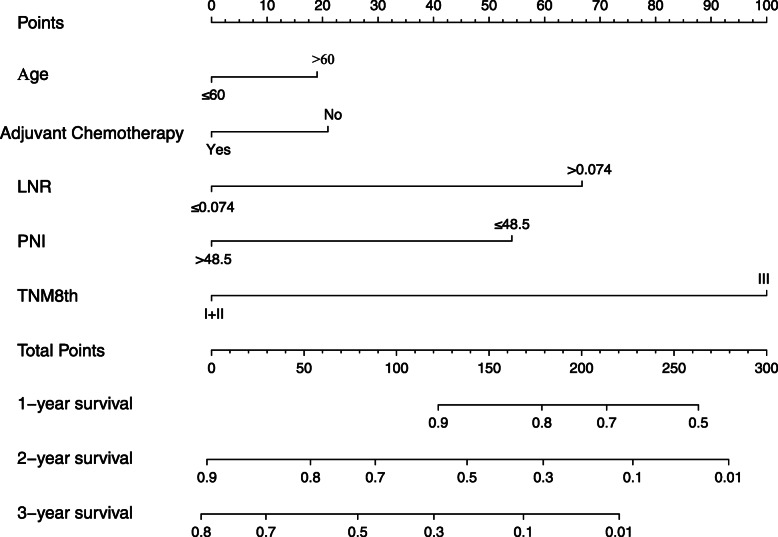
Nomogram for predicting OS of pancreatic head cancer underwent radical pancreaticoduodenectomy

The Kaplan–Meier analysis of the PNI in the primary set (Fig. 1a) and validation set (Fig. 1c) was statistically significant (*P* < 0.01). The AUC values for evaluating the performance of the PNI for 1-, 2-, and 3-year OS were 0.855, 0.875 and 0.862, respectively, in the primary set (Fig. 1b). The AUC values for evaluating the performance of the PNI for 1-, 2-, and 3-year OS were 0.739, 0.922 and 0.874, respectively, in the validation set (Fig. 1d). Additionally, the performance of the LNR for 3-year OS was 0.705 in the primary set and 0.751 in the validation set (Fig. 2). As age and postoperative adjuvant systemic chemotherapy are always regarded as predictive factors associated with pancreatic cancer prognosis, these factors were integrated into the nomogram [18–22]. The factors of age, postoperative adjuvant systemic chemotherapy, the PNI, the LNR, and the TNM 8th edition guidelines were implemented to construct a nomogram (Fig. 3).

### Comparison and validation of the nomogram

**Fig. 4:**
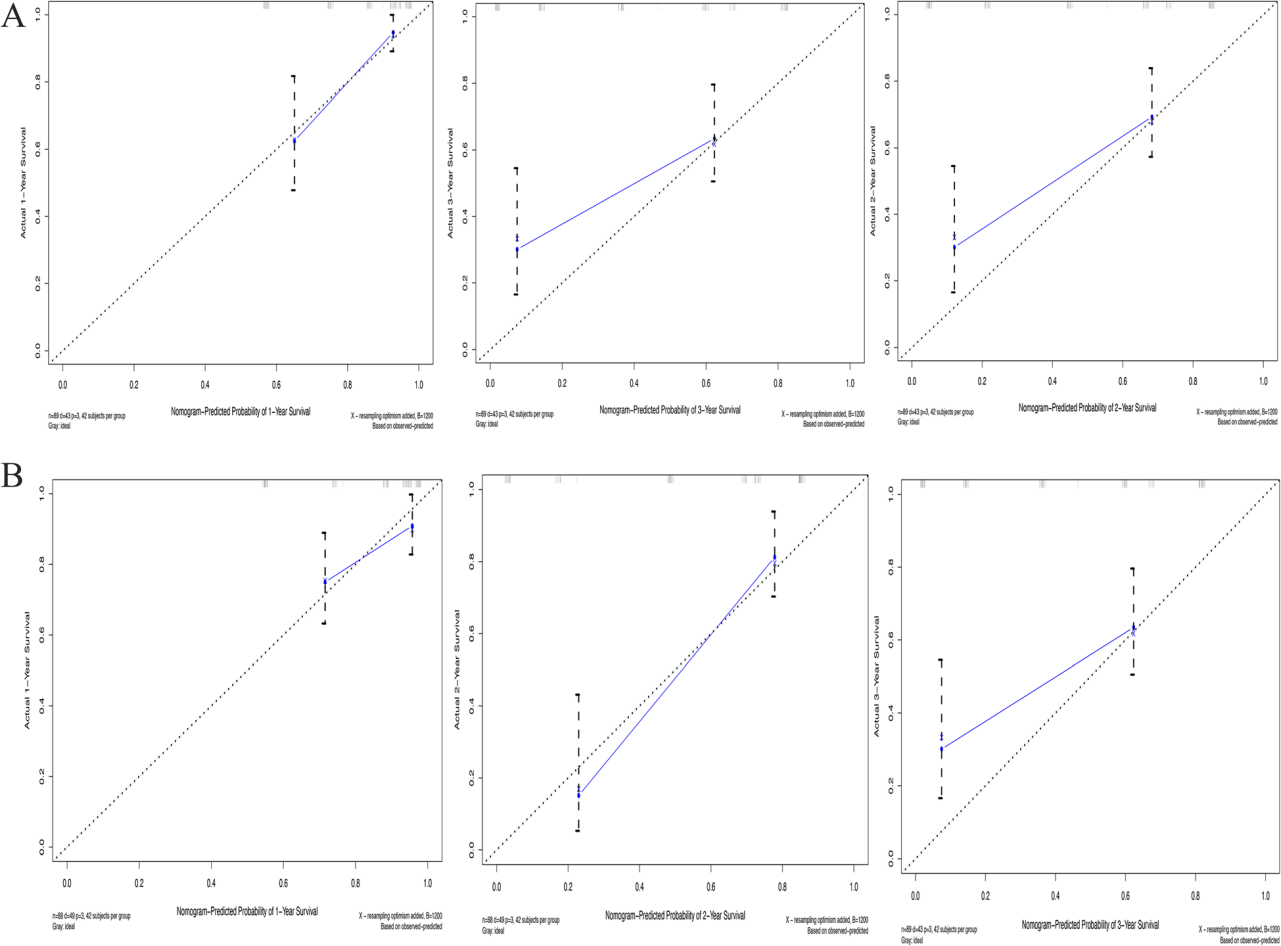
The Calibration curves for the nomogram. Nomogram for predicting 1-, 2-, and 3-year OS respectively in primary set (**a**) and validation set (**b**)

**Fig. 5:**
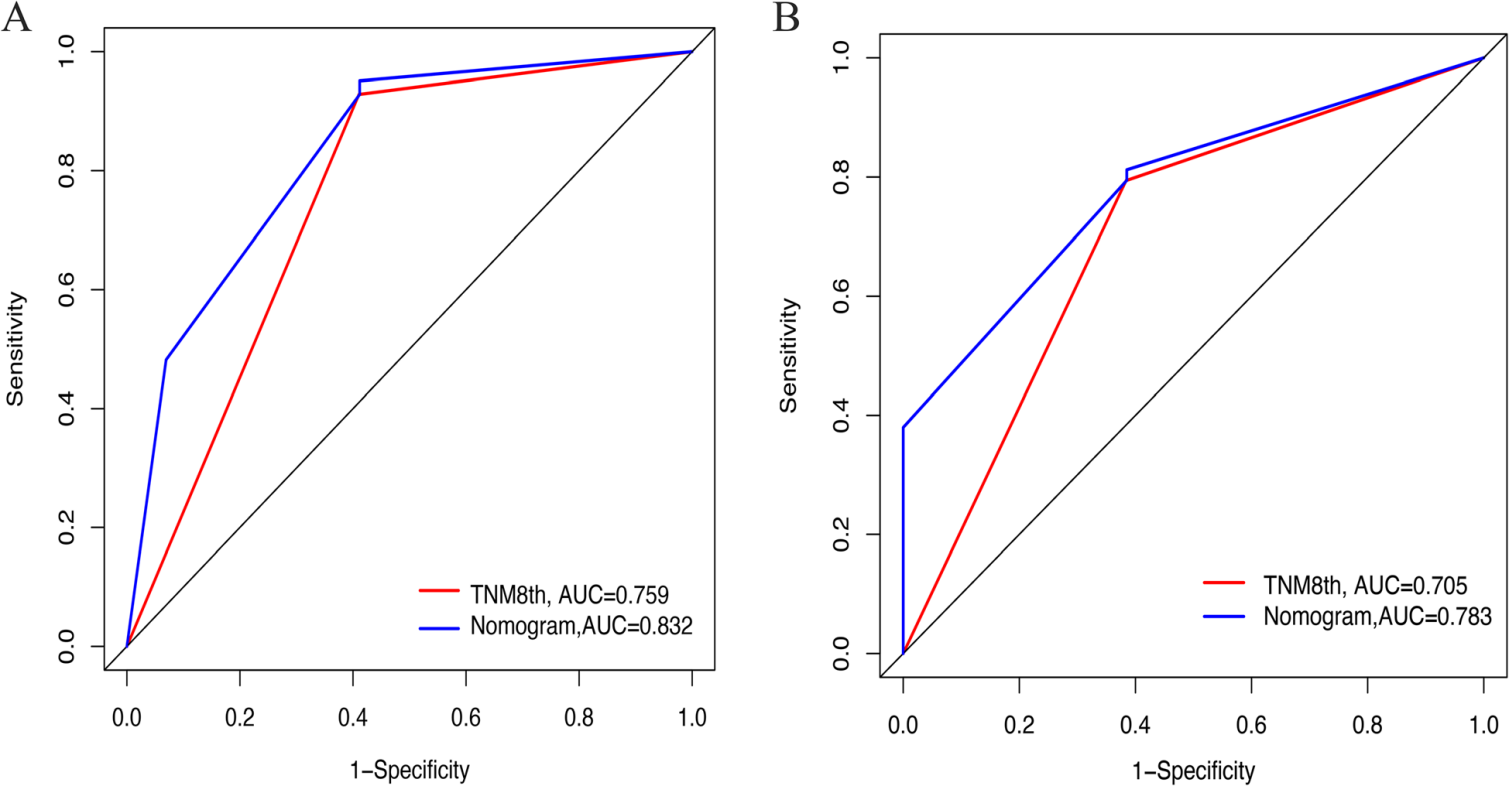
Comparison of the performance of the nomogram and AJCC stage by AUC at 1 and 3 years in primary set (**a**) and validation set (**b**)

**Fig. 6:**
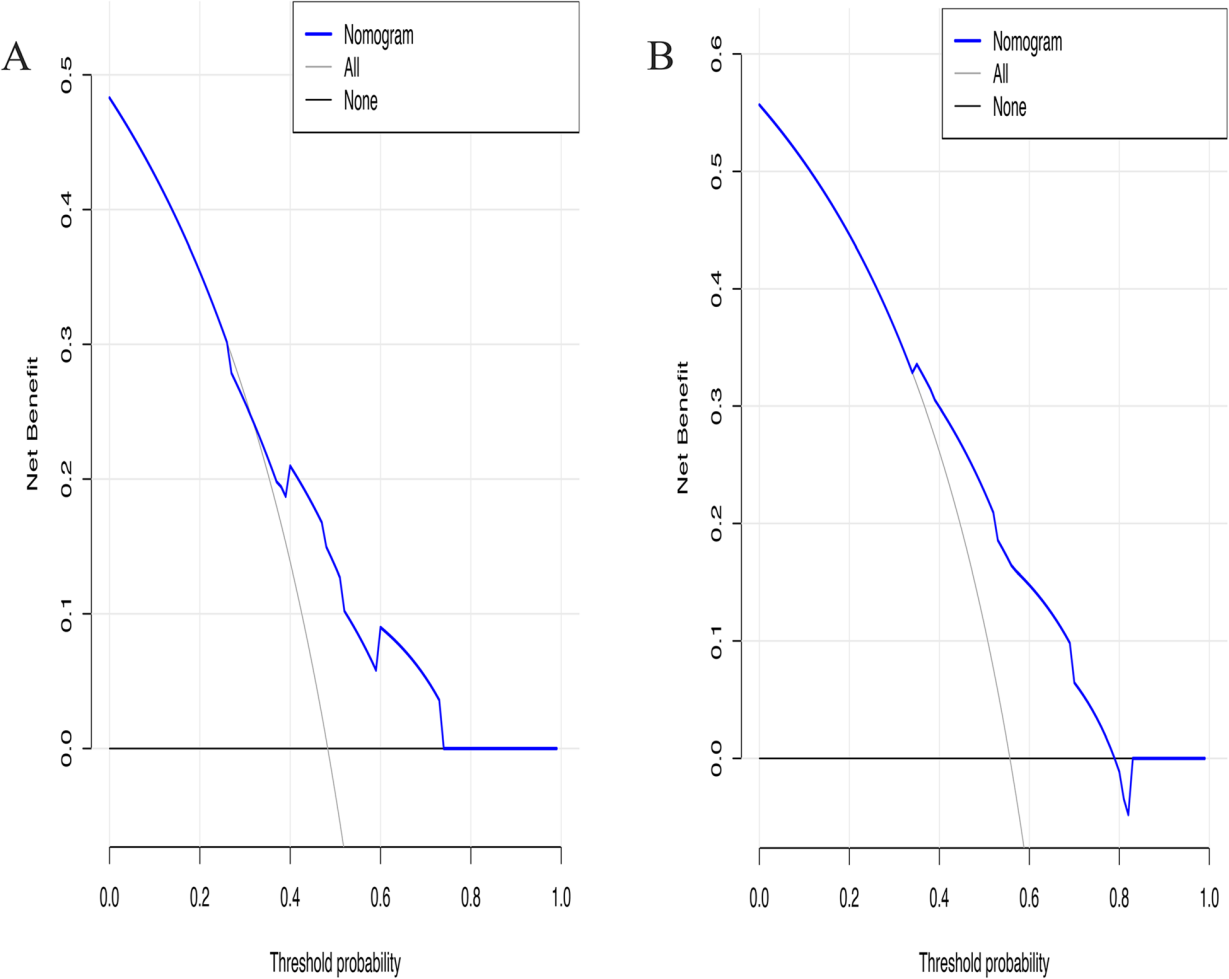
Decision curve analysis of pancreatic head cancer underwent radical pancreaticoduodenectomy in primary set (**a**) and validation set (**b**). The horizontal solid black line assumed no patients would die, and the solid grey line assumed all patients would die

The c-indexes of the nomogram were 0.799 (confidence interval (CI), 0.741–0.858) and 0.732 (0.657–0.807) in the primary set and validation set, respectively. The calibration plot for predicting 1-, 2-, and 3-year OS (Fig. 4) showed that the nomogram model performed well in the primary set and validation set. The AUCs of the nomogram at 1 and 3 years were 0.832 and 0.783, respectively, which were superior to the AJCC staging values of 0.759 and 0.705 (Fig. 5). Additionally, our study suggests that the nomogram showed a superior net benefit across a wider scale of threshold probabilities for predicting OS in the DCA (Fig. 6).

## Discussion

We retrospectively collected the clinical characteristics of 316 patients with supposed pancreatic head cancer who were admitted to the West China Hospital of Sichuan University from July 2015 to June 2017. Finally, 177 adenocarcinoma of the pancreatic head patients who underwent radical pancreaticoduodenectomy and systematic lymphadenectomy without peritoneal dissemination or distant metastases were included in the study. Subsequently, we screened out risk factors associated with the prognosis of head cancer patients who underwent pancreaticoduodenectomy to construct a nomogram. The calibration curves of the nomogram for predicting 1- and 2-year OS closely matched the ideal 45-degree line in the primary set (Fig. 4a) and validation set (Fig. 4b), meaning that the predictive power of the nomogram was significantly good. Although the calibration curves of the nomogram for predicting 3-year OS slightly deviated from the ideal 45-degree line in the primary set and validation set, notably, the c-indexes of the nomogram were 0.799 (0.741–0.858) and 0.732 (0.657–0.807) in the primary set and validation set, respectively. Additionally, the AUCs of the nomogram at 1 and 3 years were 0.832 and 0.783, respectively, which were superior to the AJCC staging values of 0.759 and 0.705 (Fig. 5). Finally, the nomogram showed a superior net benefit across a wider scale of threshold probabilities for predicting OS in the DCA. Thus, the nomogram may be an effective model for developing an optimal therapeutic schedule for adenocarcinoma of the pancreatic head patients.

Accumulating studies have been utilized to disclose the relationship between clinical characteristics and prognostic outcomes in pancreatic adenocarcinoma. For instance, lymph node metastases are considered an important factor for predicting OS in pancreatic cancer patients who undergo surgery [23–26]. However, previous studies have revealed that there are some limitations of only using the number of positive LNs to predict prognosis. The LNR is the ratio of the number of positive lymph nodes to the total number of lymph nodes dissected during surgery. In this study, the multivariate analysis suggested that the LNR was an independent factor related to OS. Additionally, previous studies have revealed that the PNI has been used as a predictive prognostic factor for hepatocellular carcinoma [27, 28], small-cell lung cancer [29, 30], nasopharyngeal carcinoma [31], gastric cancer [32], and pancreatic cancer [10]. Our study also suggested that the PNI was an independent factor for predicting OS in pancreatic head cancer patients who underwent pancreaticoduodenectomy. The published data revealed that preoperative enteral alimentation increased the serum albumin level and total lymphocyte count to improve postoperative outcomes, showing the indispensability of perioperative nutritional management [33–35]. For this reason, malnourished patients need early effective nutritional intervention to promote the treatment efficacy of resectable pancreatic head cancer. Collectively, the results of the nomogram constructed by the factors of age, postoperative adjuvant systemic chemotherapy, the PNI, and the LNR may serve as a proposal of prognostic importance for adenocarcinoma of the pancreatic head patients.

There are limitations to this study. There is a lack of sufficient predictive factor incorporation in the nomogram to provide absolute predictions. Some known factors may not have been incorporated due to the absence of numbers or observations, or there may be biomarkers that are still undisclosed. This study only used a single institution-based database; therefore, to verify the accuracy of predictive nomograms in multiple institution-based databases is necessary.

Conclusions: The nomogram may be used to predict the prognosis of radical resection for adenocarcinoma of the pancreatic head. These findings may represent an effective model for developing an optimal therapeutic schedule for malnourished patients who need early effective nutritional intervention and may promote the treatment efficacy of resectable adenocarcinoma of the pancreatic head.

## Supplementary Information

**Additional file 1.** The demographics of set.

## Data Availability

All primary data is available by sending email to correspondence author.

## Abbreviations

AUC: Area under the curve
PNI: Prognostic nutritional index
LNR: Lymph node ratio
AJCC: American Joint Committee on Cancer
CI: Confidence interval
OS: overall survival
