# Supplementary material for: Prognostic Nomogram for patients undergoing radical Pancreaticoduodenectomy for adenocarcinoma of the pancreatic head
Source: BMC Cancer. 2021 May 27;21:624. doi: 10.1186/s12885-021-08295-5 (PMC8161963; doi:10.1186/s12885-021-08295-5)
Supplement: Supplementary file 1 — Additional file 1. The demographics of set. [file 12885_2021_8295_MOESM1_ESM.pdf]

S1 The demographics of set

Variables	All patients (n = 177)	Primary set (n=89)	Validation set (n=88)
CA19-9(u/ml)			
≤37	31	20	11
(37 222]	51	21	30
>222	95	48	47
CEA(ng/ml)			
≤5	84	47	37
>5	93	42	51
Tbil(μmol/L)			
≤37	78	35	43
(37 222]	53	35	18
(222-296]	26	9	17
>296	20	10	10
Age			
≤60	84	42	42
>60	93	47	46
Chemotherapy			
No	158	77	81
Yes	19	12	7
Sex			
Female	68	36	32
Male	109	53	56
TNM8th			
I+II	116	56	60
III	61	33	28
LNR			
≤0.074	64	29	35
>0.074	113	60	53
PNI			
≤48.5	90	41	49
>48.5	87	48	39
